# Complete genome sequence of fig leaf mottle-associated virus 2

**DOI:** 10.1007/s00705-025-06262-0

**Published:** 2025-03-11

**Authors:** Rachelle Bester, Shannon Goodchild, Hans J. Maree

**Affiliations:** 1 Department of Genetics, Stellenbosch University, Private Bag X1, Matieland, 7602 South Africa; 2 Citrus Research International, PO Box 2201, Matieland, 7602 South Africa

## Abstract

The complete genome sequence of fig leaf mottle-associated virus 2 (FLMaV2), a positive-sense single-stranded RNA virus with a genome length of 16,925 nucleotides, was determined via RT-PCR and rapid amplification of cDNA ends (RACE). Its genome organization resembles those of little cherry virus 2 (LChV2) and yam asymptomatic virus 1 (YaV1), both of which are currently classified as members of the genus *Ampelovirus* but differ from typical ampeloviruses. Phylogenetic analysis based on amino acid sequences of the RNA-dependent RNA polymerase and coat protein of members of the family *Closteroviridae* showed that FLMaV2 clustered with LChV2 and YaV1. Because of their unusual genome organization, FLMaV2, LChV2, and YaV1 might represent a distinct genus within the family *Closteroviridae*.

**Supplementary Information:**

The online version contains supplementary material available at 10.1007/s00705-025-06262-0.

The family *Closteroviridae* includes non-enveloped, filamentous viruses with a 13- to 19-kb positive-sense RNA genome that is either translated directly to make polyproteins or transcribed to make subgenomic RNAs [1]. The family includes seven genera [2], one of which is the genus *Ampelovirus*, which has 13 classified species (https://ictv.global/taxonomy). Ampeloviruses share a conserved genome organization, featuring a two-gene module (ORF1a–ORF1b) encoding replication proteins and a five-gene module (small hydrophobic protein, heat shock protein 70 homologue [HSP70h], ~60-kDa protein, coat protein [CP], and minor coat protein [CPm]) [3, 4]. Fig leaf mottle-associated virus 2 (FLMaV2), first identified in Algeria [5] and later reported in Syria, Egypt, Lebanon, Tunisia, Albania, Turkey, and South Africa [6, 7], has been proposed to be an ampelovirus but remains unclassified due to the lack of a complete genome sequence. Only a partial 5868-nt sequence (FJ473383) is available, and a complete genome sequence is essential for its accurate classification and characterization.

In July 2021, fig leaves with mottling and mosaic patterns from three garden trees in the Western Cape were analysed using high-throughput sequencing (HTS) as described previously [7], yielding a 15,206-nt contig with a high degree of sequence similarity to FLMaV2 (FJ473383.1). This draft genome sequence was used to design primers to produce overlapping amplicons (Supplementary Information S1). In 2023, additional fig leaf samples from the same garden were collected, and RNA was extracted [8, 9] and screened for FLMaV2 using primer set 16 (Supplementary Information S1). Complementary DNA (cDNA) was synthesized from total RNA using random hexamer primers (Promega) and Maxima Reverse Transcriptase (Thermo Fisher Scientific), following the manufacturer’s instructions. PCR was then performed using Phusion HF polymerase (New England Biolabs) and genome-specific forward and reverse primers (IDT) (Supplementary Information S1), following the manufacturer’s instructions. DNA samples containing the amplicons of interest were sent to the Central Analytical Facility (CAF) of Stellenbosch University for bidirectional Sanger sequencing using the same primers that were used for amplification. Five of the eight trees were positive for FLMaV2 by RT-PCR using primer set 16, and pairwise comparisons of the Sanger-sequenced amplicons from the different tree samples revealed a 96.45–100% identity to the HTS draft genome sequence. The sample with the most divergent sequence of FLMaV2 compared to the draft genome sequence was selected for genome confirmation. RNA from sample F8D3 was amplified using primer sets 1-19 and Phusion HF polymerase (New England Biolabs) (Supplementary Information S1). The amplicons were sequenced by the Sanger method as described above. To amplify the 5’ end of the genome, a SMARTer rapid amplification of cDNA ends (RACE) 5’ kit (Takara Bio) was used according to the manufacturer’s instructions. Plasmid DNA from eight clones was sent to CAF at Stellenbosch University for bidirectional sequencing using M13 primers. For the 3’ end, total RNA was polyadenylated using *E. coli* poly(A) polymerase (New England Biolabs) according to manufacturer’s instructions and used for reverse transcription with an oligo d(T) primer (primer 21, Supplementary Information S1) to generate cDNA. The cDNA was subjected to thermal cycling using Phusion HF polymerase, an oligo d(T) primer, and a specific forward primer (primer 22, Supplementary Information S1). A DNA sample containing the amplicon of interest was sent to CAF at Stellenbosch University for Sanger sequencing using primer 22. The complete genome sequence of isolate F8D3 (16925 nt) was assembled from the overlapping amplicons using CLC Main Workbench 7 (QIAGEN) (PQ727362). ORF Finder (https://www.ncbi.nlm.nih.gov/orffinder/) was used to predict open reading frames (ORFs).

The partial FLMaV2 sequence that had been submitted previously to NCBI GenBank (FJ473383.1) shared 83.85% nucleotide (nt) sequence identity with the corresponding region of the complete FLMaV2 genome sequence from this study (PQ727362), suggesting that the South African FLMaV2 isolate is a divergent variant. This was further supported by analysis of a 1179-bp amplicon spanning the ORFs encoding the HSP70 and HSP90 homologues. Sample F8D3 exhibited the greatest genetic divergence, with 96.62% nt sequence identity to the other four FLMaV2 sequences, which were 100% identical over 1064 nt. F8D3 shared 82.07% nt sequence identity with FJ473383.1, while the other four sequences exhibited 81.13% identity.

The FLMaV2 isolate from this study has the same genome organization as FJ473383.1 (Fig. 1). The 5’ UTR is 1005 nt in length and is followed by nine putative ORFs in the positive orientation and a 3’ UTR of 231 nt. This organization resembles that of little cherry virus 2 (LChV2) and yam asymptomatic virus 1 (YaV1), which are classified as ampeloviruses [10, 11] but are distinct from other members of this genus. For FLMaV2, LChV2, and YaV1, the L-Pro and AlkB domains in ORF1a were not detected, and several 3’ ORFs (e.g., p21, p20A, p20B, p4, and p7) are absent. Notably, the conserved ampelovirus five-gene module (p5, Hsp70h, p55, CP, CPm) is disrupted by an additional p21 gene before the CP gene in these viruses (Fig. 1). A similar-sized ORF (p18) that was in the -1 reading frame of both YaV1 and LChV2 (Fig. 1) could not be identified in FLMaV2. However an ORF from position 383 to 511 in the -1 reading frame was predicted to have coding potential when analyzed using GeneMark [12]. Due to a lack of similarity to any sequences in YaV1 and LChV2 or any other sequence in the GenBank database, it was not annotated as an ORF.

**Fig. 1:**
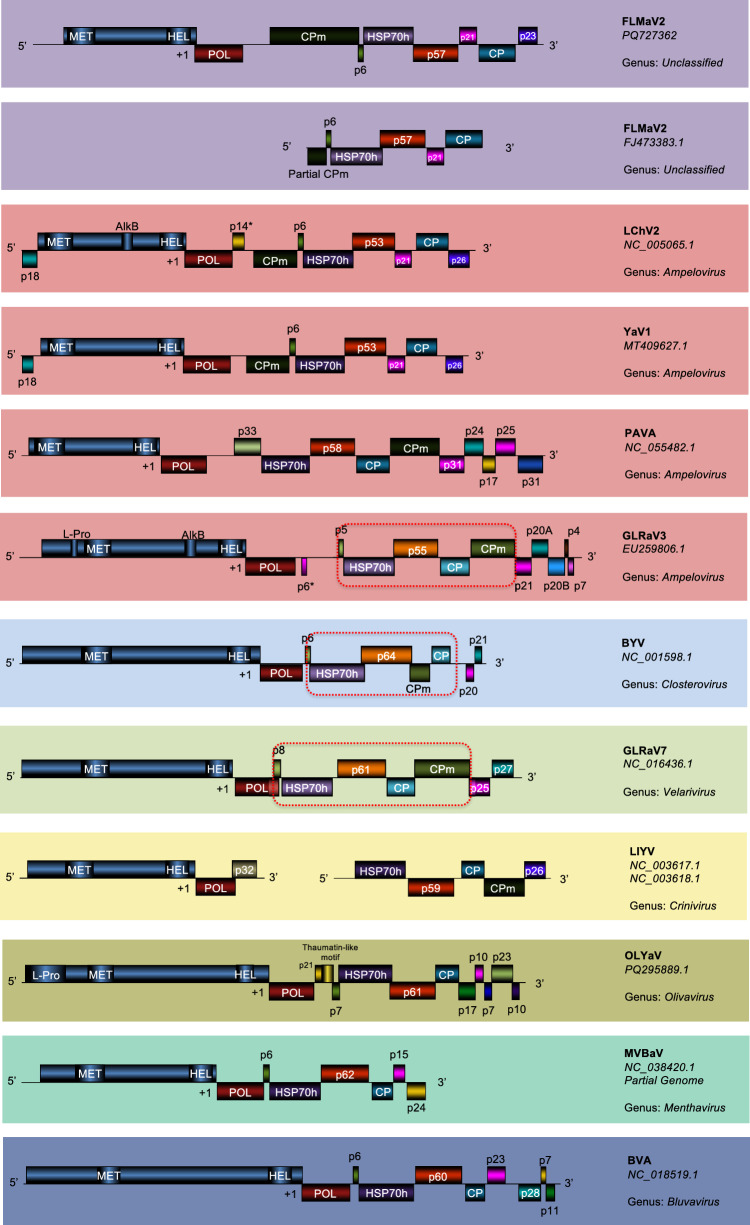
Genome organisation of fig leaf mottle-associated virus 2 (FLMaV-2) from South Africa (PQ727362) in comparison to the partial NCBI sequence of FLMaV-2 (FJ473383.1), little cherry virus 2 (LChV-2), yam asymptomatic virus 1 (YaV1), pistachio ampelovirus A, and representative members of each of the seven genera of the family *Closteroviridae*. The following viruses were selected as representatives of their genus: grapevine leafroll-associated virus 3 (GLRaV-3) for the genus *Ampelovirus*, beet yellow virus (BYV) for the genus *Closterovirus*, grapevine leafroll-associated virus 7 (GLRaV-7) for the genus *Velarivirus*, lettuce infectious yellows virus (LIYV) for the genus *Crinivirus*, olive leaf yellowing-associated virus (OLYaV) for the genus *Olivavirus*, mint vein banding-associated virus (MVBaV) for the genus *Menthavirus*, and blueberry virus A (BVA) for the genus *Bluvavirus*. The conserved five-gene module in members of the genera *Ampelovirus*, *Closterovirus* and *Velarivirus* is indicated by a red block. "*" indicates that the start site of p14 of LChV2 was not determined, and this gene may be expressed by readthrough of an upstream open reading frame or initiation from a non-AUG start codon. The p6 of GLRaV3 is only present in some isolates of this virus.

To determine the phylogenetic relationship of FLMaV2 within the family *Closteroviridae*, multiple sequence alignments of the RNA-dependent RNA polymerase (RdRp) and CP regions were made using MAFFT version 7 [13], and maximum-likelihood (ML) phylogenetic trees were constructed using IQ-Tree [14], using automatic model selection and 1000 ultra-fast bootstrap replicates. The resulting trees were visualized using FigTree v1.4.4. Phylogenetic analysis based on amino acid sequences of RdRp (Fig. 2) and CP (Supplementary Information S2) showed that FLMaV2 clustered with LChV2 and YaV1, in a separate clade from the other members of the genus *Ampelovirus*, with the exception of pistachio ampelovirus A (PAVA), whose RdRp sequence clustered with LChV2, YaV1, and FLMaV2 (Fig. 2) while its CP sequence was most similar to that of GLRaV-13 (Supplementary Information S2).

**Fig. 2:**
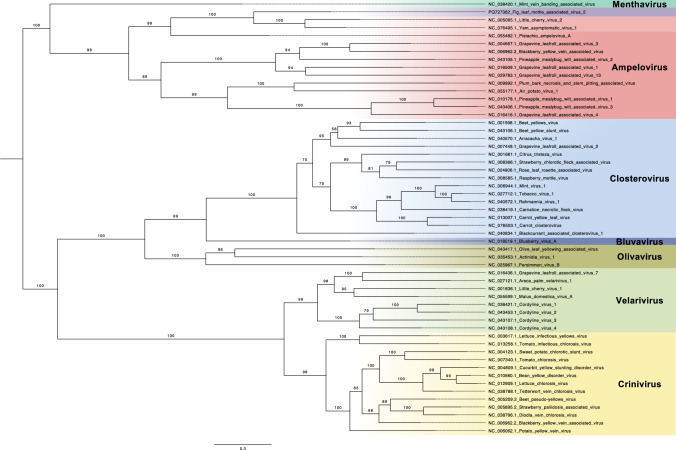
Maximum-likelihood (ML) phylogenetic tree based on a multiple alignment of RNA-dependent RNA polymerase (RdRp) amino acid sequences predicted using the NCBI ORF Finder program (allowing any sense codons) from each of the GenBank reference sequences of the family *Closteroviridae.* Model selection was determined automatically (LG+F+I+G4), and 1000 replicates were performed for ultra-fast bootstrap support. The final tree was visualised using FigTree, and midpoint rooting was applied. Branch lengths are indicative of the number of substitutions per site.

The FLMaV2 coat protein (CP) has an estimated molecular weight of 44.15 kDa, making it significantly larger than the typical ampelovirus CP (28-36 kDa) (https://ictv.global/report/chapter/closteroviridae/closteroviridae). Similarly, LChV2 (39.23 kDa), YaV1 (36.17 kDa), and PAVA (39.32 kDa) also have larger CPs. While the genome organization of PAVA aligns with that of most ampeloviruses, its larger CP and sequence similarity to FLMaV2 in its RdRp and CP indicate that it is also not a typical member of the genus *Ampelovirus*.

This is the first report of the complete genome sequence of FLMaV2, which, despite sharing similarities with members of the genus *Ampelovirus*, exhibits significant differences that challenge its classification within this genus. Differences in their genome organization and phylogenetic relationships place FLMaV2, LChV2, and YaV1 in a separate cluster, suggesting a need to revisit the taxonomic framework of the family *Closteroviridae*. We propose establishing a new genus to accommodate FLMaV2, LChV2, and YaV1, reflecting their evolutionary distinctiveness.

## Supplementary Information

Below is the link to the electronic supplementary material.

Supplementary file1 (PDF 112 KB)

Supplementary file2 (PDF 111 KB)

## Data Availability

The datasets generated and/or analysed in the current study are available from the corresponding author on reasonable request.
