# Supplementary material for: Complete genome sequence of fig leaf mottle-associated virus 2
Source: Arch Virol. 2025 Mar 11;170(4):72. doi: 10.1007/s00705-025-06262-0 (PMC11897069; doi:10.1007/s00705-025-06262-0)
Supplement: Supplementary file 1 — Supplementary file1 (PDF 112 KB) [file 705_2025_6262_MOESM1_ESM.pdf]

Supplementary Information (SI) 1

Unveiling the genome of fig leaf mottle-associated virus 2
Archives of Virology

R Bester[1,2], S Goodchild[1] and HJ Maree[1,2†]
[1] Department of Genetics, Stellenbosch University, Private Bag X1, Matieland, 7602, South Africa
[2] Citrus Research International, PO Box 2201, Matieland, 7602, South Africa

†Corresponding author: HJ Maree
email: hjmaree@sun.ac.za

SI 1 Properties of the primers used to generate the sequence data of the South African genome of FLMaV2 F8D3 (PQ727362).

Primer Pair	Primers names	Primer sequences (5'-3')	Amplicon size (bp)	Annealing temperature (°C)	Extension time (s)
1	FLMaV2_5F	GGATGAAGGGGAGGTGGAAG	1122	60	45
	FLMaV2_1127R	CAGTAAAGACGACCGCCGCT			
2	FLMaV2_823F	AGGTAGCTGACACTCGTGATT	741	50	30
	FLMaV2_1564R	ACCAGTGCTTCCGAAACAACA			
3	FLMaV2_1023F	CCCAAGGAAGGAGAGAAAACC	1174	60	45
	FLMaV2_2197R	GCAGTGGCATCGCAAATCAAAG			
4	FLMaV2_2063F	TGAGGTTGTTGAGAGAGAGAC	1217	60	45
	FLMaV2_3280R	CCTTGAGGATAGCACAGATTTC			
5	FLMaV2_2788F	AGATTACCCTCGAAGACACACC	834	55	30
	FLMaV2_3622R	CCAATCCCTTCAGTCATCCTC			
6	FLMaV2_3161F	GATAGATGGTAAGCGGATGC	1195	60	45
	FLMaV2_4356R	GTTGATACTGGGACGGTGAT			
7	FLMaV2_4187F	GGCGTTTTGCGAATGTTGTTGC	1215	60	45
	FLMaV2_5402R	ATCAACAGAGGGACAAAGCC			
8	FLMaV2_4992F	TAGAAATCAGGACCTCGGGCG	1023	55	45
	FLMaV2_6015R	AAAGTCAGGACAACCCCAGG			
9	FLMaV2_5277F	GTGTTAGCCCTTTTCTGTCTC	1199	55	60
	FLMaV2_6475R	CCCCACTACTGTTTTTCACTTC			
10	FLMaV2_6171F	TATCATTGTTGGTGGTGTTCGT	1028	55	45
	FLMaV2_7199R	ACGCTCTGTGGACCTACAAAA			
11	FLMaV2_6362F	TAGGTTGGGAGATTCAGTTCAG	1271	60	45
	FLMaV2_7633R	TAGACACATAGACTTCCACCG			
12	FLMaV2_7513F	CATCGGCGGGAGTTGAATTTG	1223	60	45
	FLMaV2_8736R	CTTTCCAGACACCACCAATCC			
13	FLMaV2_8543F	CTGGTTTGATGGAGAAGGATGTG	1249	60	45
	FLMaV2_9792R	TGTTTACTCCTACCTCCTGC			
14	FLMaV2_9541F	AAGGGCGAGAGTATGAGGTGGA	630	55	30
	FLMaV2_10171R	GGGGCCGCAACCACACATAAA			
15	FLMaV2_9587F	AGTGGCGATAGGAGGAATAG	1149	53	45
	FLMaV2_10736R	AACTCTCCCCGATGATTTTGG			
16	FLMaV2_10532F	AACGGGCACAATGACAATACC	1204	60	45
	FLMaV2_11742R	GCATTCTCACCTACAGTCAGT			
17	FLMaV2_11536F	TGTGGGGGATGTTAGTATGC	1178	60	45
	FLMaV2_12714R	TGTCAGAACGTCAGGCAATTCG			
18	FLMaV2_12488F	CCTCAAAATCAACGGACGTGG	1258	60	45
	FLMaV2_13746R	GCCGAACTAAACACACCAGG			

Primer Pair	Primers names	Primer sequences (5'-3')	Amplicon size (bp)	Annealing temperature (°C)	Extension time (s)
19	FLMaV2_13558F	ACAACAATCTCAAACTGGTGCG	1233	50	45
	FLMaV2_14791R	TTGTCGCCGTATCTTCTTATGG			
20	FLMaV2_RACE_1353R	GATTACGCCAAGCTTTCCCCTGACTGGACACGACACGACC		68	120
21	GVA-dT(17)	TACGATGGCTGCAGTTTTTTTTTTTTTTTTT			
22	FLMaV-2_14658F	GTGTGTAAGACCATTTTGACTG		55	30
