# Supplementary material for: Complete genome sequence of fig leaf mottle-associated virus 2
Source: Arch Virol. 2025 Mar 11;170(4):72. doi: 10.1007/s00705-025-06262-0 (PMC11897069; doi:10.1007/s00705-025-06262-0)
Supplement: Supplementary file 2 — Supplementary file2 (PDF 111 KB) [file 705_2025_6262_MOESM2_ESM.pdf]

Supplementary Information (SI) 2

Unveiling the genome of fig leaf mottle-associated virus 2

Archives of Virology

R Bester[1,2], S Goodchild[1] and HJ Maree[1,2†]

[1] Department of Genetics, Stellenbosch University, Private Bag X1, Matieland, 7602, South Africa

[2] Citrus Research International, PO Box 2201, Matieland, 7602, South Africa

†Corresponding author: HJ Maree

email: hjmaree@sun.ac.za

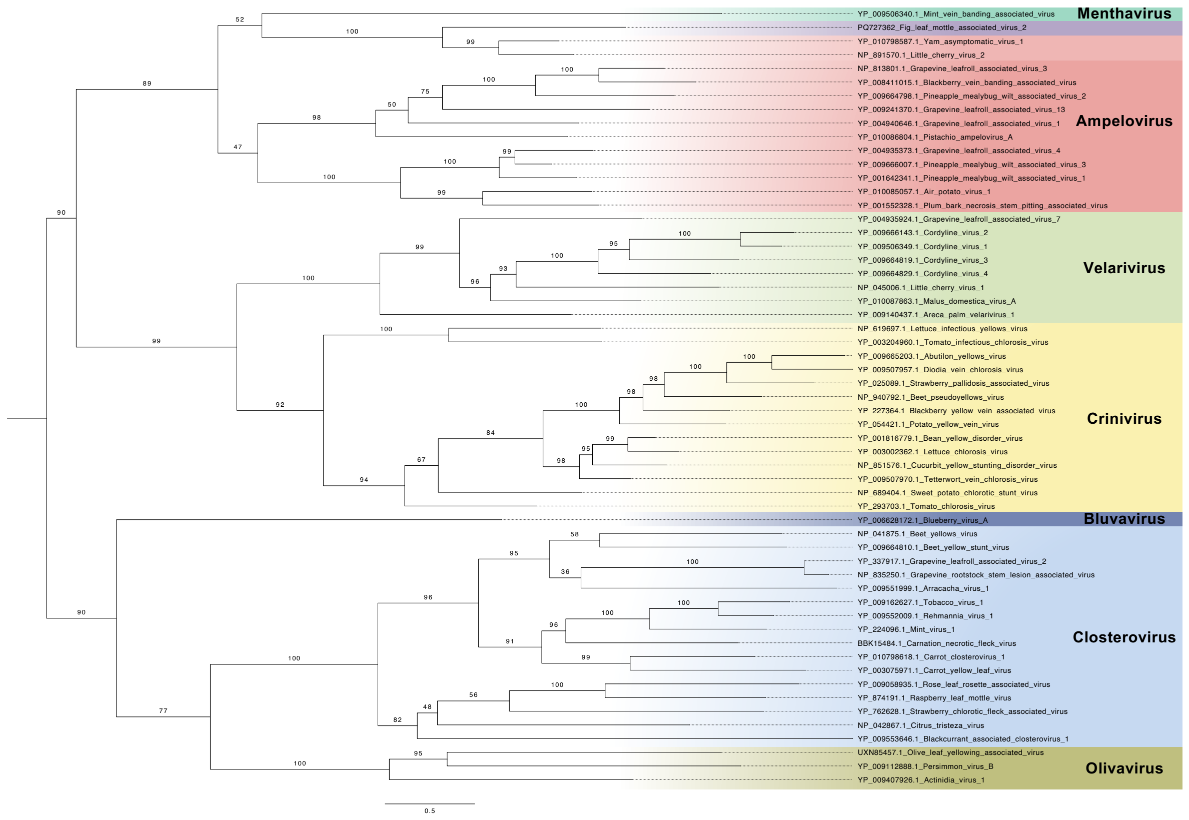

SI 2 A Maximum Likelihood (ML) tree inferred from a multiple sequence alignment of the coat protein (CP) amino acid sequences from each of the GenBank reference sequences from the family *Closteroviridae.* Model selection was automatically determined (LG+F+I+G4), and 1000 replicates were performed for Ultra-Fast Bootstrap support. The final tree was visualised using FigTree and midpoint rooting was applied. Branch lengths are indicative of substitutions per site.
